# Evaluation of an Age-Friendly City and Its Effect on Life Satisfaction: A Two-Stage Study

**DOI:** 10.3390/ijerph16245073

**Published:** 2019-12-12

**Authors:** Raquel Flores, Antonio Caballer, Ana Alarcón

**Affiliations:** Department of Developmental, Educational and Social Psychology and Methodology. Universitat Jaume I, Castellón 12071, Spain; caballer@uji.es (A.C.); alarcon@uji.es (A.A.)

**Keywords:** demographic ageing, active ageing, age-friendly cities, life satisfaction

## Abstract

Due to the ageing of the world’s population, age-friendly cities are emerging to promote active ageing by optimising opportunities for health, participation and safety, in order to improve the quality of life of older people. Despite initiatives in different countries, there is a lack of empirical research exploring their impact on older people. The objective of this study is to evaluate an age-friendly city by analysing its relationship with life satisfaction, taking into account the age cohort variables of the elderly and whether they live alone or with someone else. A two-stage study, in which 66 subjects participated in the qualitative analysis (focus groups) in Stage I and 203 of the quantitative analysis (survey methodology) or Stage II. Despite the differences found in the different groups of elderly persons, for all of them the domains of outdoor spaces and buildings, and community support and health services, are significantly related to life satisfaction, with the latter showing itself to be a predictor of such satisfaction. It is important to take into account the different groups of elderly persons, so as to be able to establish suitable specific actions. This study aims to make a contribution to the development of public policies that influence the life satisfaction of the elderly.

## 1. Introduction 

The ageing of the world’s population entails changes in all sectors of society. This increase in the elderly population is due to the conjunction of several factors, such as increased life expectancy and advances in health systems, public hygiene and sanitation [1]. In 2017, the world population of people over 60 years of age was 962 million, and continues to grow at an annual rate of 3%, so this figure is expected to double by 2050 and triple by 2100 [2]. 

Faced with this situation, the paradigm of active ageing has become increasingly relevant in contemporary global gerontological, political and social discussions [3,4], and is considered the most important policy response to demographic ageing [5,6].

The term active ageing is understood as a process of optimizing opportunities to enhance physical, social and mental well-being throughout the life cycle, in order to extend healthy life expectancy [7]. At present, there is no consensus on the criteria for establishing active ageing [8,9]. A systematic review on this subject [10] shows the heterogeneity of the criteria used in this respect, some of the most salient being the physiological state (health, functional status or longevity), well-being (affectivity and life satisfaction), active involvement in social life (support systems, social activities), personal resources (coping) and extrinsic factors (environment and economic level). Thus, various studies on active ageing establish a relationship among the characteristics of the physical environment, and the health and the participation of the elderly [11,12]. 

To put the paradigm of active ageing into practice, the World Health Organisation (WHO) has established the Age-Friendly Cities and Communities initiative, which promotes active ageing by optimising health, participation and safety opportunities in order to improve people’s quality of life as they age [13,14]. However, there is no universally accepted definition of what is meant by an age-friendly city. The concept is linked to various aspects, such as the physical and social environment [15], rights and citizenship [13], and adequate housing and transport [16]. The initiative proposed by the WHO [17] follows the methodology developed in the Vancouver Protocol [18] and is widely recognised as a global movement around the world [19,20]. This protocol uses a bottom-up participatory approach based on elderly persons’ experience in order to identify the characteristics of an age-friendly city and to assess a range of built, social and service environments in eight domains: outdoor spaces and buildings, transportation, housing, social participation, respect and social inclusion, civic participation and employment, communication and information, and community support and health services [17,21]. These areas are related to elderly persons’ life satisfaction [22,23], understood as a cognitive component of well-being that refers to an individual’s evaluation of his or her life [24]. Thus, various studies show the relationship between life satisfaction and the social resources the person has available to them [25,26], social functioning [27], participation and integration [28,29], physical characteristics of the environment [30,31] and perception of safety [32,33].

In 2010, the WHO launched the Global Network for Age-Friendly Cities and Communities [14,34]. This Network currently has 533 member cities and communities in 37 countries, covering over 179 million people around the world [35]. Cities joining the Network are required to commit to a five-year cycle of continually assessing and improving their age-friendliness [14,36].

In recent years, there has been a growing interest in developing a new agenda for the age-friendly cities movement [13,37], increasing the number of reviews on the subject [21,38,39,40] and stepping up research on the evaluation of age-friendly cities in different countries [41,42,43,44]. However, the methodology used in the studies has met with some criticism [20] which points out that the surveys conducted may not be well suited to reflect the views of the elderly [45,46]. Despite initiatives to improve the adequacy of cities for the elderly, there is a paucity of empirical research exploring their role in elderly persons’ life satisfaction [47,48] and few studies assessing how the characteristics of cities can influence the well-being of this age group [49,50].

Therefore, the present study aims to (1) evaluate an age-friendly city with a two-stage study approach, so as to integrate research results with policies in order to improve the quality of life of the elderly in urban areas; (2) evaluate the relationship between life satisfaction and the specific domains of an age-friendly city, taking into account different age cohorts of elderly persons; and (3) evaluate the relationship between life satisfaction and the specific domains of an age-friendly city, taking into account whether the elderly persons live alone or with someone else.

## 2. Materials and Methods

The research was conducted using a two-stage study, which combines qualitative and quantitative analyses. The first stage was to use a qualitative methodology to explore and generate data. Then, in the second stage, a cross-sectional survey (quantitative methodology) was used, that is, a sequential exploratory design was followed. This research has complied with the ethical standards of the 1964 Declaration of Helsinki and its later amendments.

### 2.1. Participants

The qualitative study was conducted by means of focus groups. A total of eleven focus groups were conducted, seven groups with older adults of different ages (60–74 years; 75 and over) and socio-economic status (low; medium) (Table 1), and four groups of service providers in each of the following categories: municipal or regional public service professionals, entrepreneurs and traders, and voluntary organizations. In this paper, we only take into account the information from focus groups conducted with older adults (*n* = 66). When recruiting the participants, the aim was to have heterogeneous groups, whose members could freely exchange their views on issues related to the different domains. The selection of the participants of the discussion groups was carried out by Social Services professionals of Castelló City Council. Groups were selected according to two age groups (60–74 and 75 and over) and to socio-economic status (SES). Following recommendations from the Vancouver Protocol, selection by SES was based on the SES of the neighbourhood where the participants reside, not on the income of individual participants, to avoid asking potentially intrusive questions about personal income. 

The quantitative study was based on a survey of a representative sample of community residents aged 60 or over in Castelló (Spain), consisting of 203 people (112 women and 91 men; mean age = 74.4; range = 60–91). The confidence level was 95.5% (two sigmas), P = Q and an error of 7%. Table 2 shows the characteristics of the participants. Participants in the quantitative study were recruited from community centres in Castelló City using a cluster sampling. The sample was selected through a two-stage sampling procedure: clusters for the selection of the first-level units (social centres) and quotas, according to gender and age group (60–74 years; 75 and over), for the selection of second-level units (individuals). 

### 2.2. Measurements

#### 2.2.1. Qualitative

The methodology used was the Vancouver Protocol [17,18,20]. The foundation of the Vancouver Protocol was the WHO concept of active aging, and it proposed a qualitative assessment of eight domains [51]. The specific methodology includes: documentation of the profile of the city and the older adult population, and focus groups with older persons, differing by age and socio-economic status, as well as with a group of caregivers of elderly persons and a group of service providers from the public, private and voluntary sectors. 

#### 2.2.2. Quantitative

The Age-Friendly City Questionnaire (AFCQ) based on the Checklist of Essential Age-friendly City Features [52,53] was used. The eight domains were assessed using 44 items. Participants were asked to rate their views on each item using a 5-point Likert-type scale, ranging from 1 (“strongly disagree”) to 5 (“strongly agree”). Responses for each domain were averaged as a mean score. The reliability (Cronbach’s alpha) for AFCQ was 0.90. An item from the World Values Survey was used to measure life satisfaction: How satisfied are you with your life as a whole these days?” Participants rated the item on a 10-point Likert-type scale ranging from 1 (“completely dissatisfied”) to 10 (“completely satisfied”).

### 2.3. Data Collection and Analysis

Qualitative: All focus groups were led by three people: one of the authors and two professional members of staff from the City Council. At the beginning of a focus group, rules of equal turn-taking and respect were emphasized. Each of these focus groups lasted about two and a half hours, with a half-hour coffee break. Interaction in the groups was encouraged. Participants were first introduced to the concept of an age-friendly city and its related purposes were explained. They were then asked to rate how much Castelló has achieved according to the WHO checklist. Finally, they were invited to identify the common concerns that hinder related development and their views were noted. All those who finally participated in the study signed a written informed consent document, which included the title of the study, the purpose of the research, and the study procedures.

For data analysis, the researchers read the opinions transcribed in the different discussion groups and classified the main ideas presented according to the topics they addressed. 

Quantitative: Participants were introduced to the background of the study. The survey was conducted by trained interviewers and research assistants, using the Age-Friendly City Questionnaire (AFCQ). The interviews took place in different social centers of the city, targeted at elderly persons. The data analysis was performed with the statistical package IBM SPSS® Statistics version 23 (IBM Corporation, New York, NY, USA). The mean scores were compared in order to determine the score for each domain and the life satisfaction of the elderly persons, according to their age cohort [54] or whether they live alone or with someone else. In order to establish the relationship between the different domains of the city and life satisfaction, correlation analyses and multiple linear regression were run. The regression analysis was conducted after controlling the effect of the demographic variables age and gender. The predictors of life satisfaction were formed into two blocks and analyzed hierarchically using the enter method. The first block of variables included age and gender and were evaluated as statistically controlled variables of satisfaction. The second block of variables included age-friendly city domains, with age and gender adjusted.

## 3. Results 

### 3.1. Qualitative

Regarding the socio-demographic data, as can be seen in Table 1, 59% of the 66 participants in the discussion groups were ‘younger elderly’ persons, and the remaining 41% were over the age of 75. The sample is practically balanced with respect to gender, with a greater number of the elderly having a low socio-economic level (71%). As can be observed, 45.5% have a primary education, followed by 33.3% with university studies and 21.2% having secondary studies. The majority of them (81.8%) live with someone else, mostly with their spouse (72.2%).

The following are the general results obtained for each of the eight areas analyzed in the different focus groups [55]:

Outdoor spaces and buildings. Aspects that stand out include the importance of enjoying a pleasant, clean environment; green spaces; having places to stop and rest (street furniture and public toilets); suitable pavements (surface conservation, kerb height, wide pavements and slope inclination); safe pedestrian crossings (appropriate location and well indicated); and accessible urban elements. They consider these as fundamental aspects in order to feel safe.

Transportation. In the different groups, great importance was attached to the friendly attitude of the drivers of public transport vehicles and to responsible driving (to avoid falls or blows). The correct location and condition of stops are considered essential, with special emphasis on the need for adequate visibility and information about timetables and routes, and the proximity of the routes to services frequently used by the elderly. Likewise, they consider it necessary to provide more adequately adapted transportation (use of ramps, platforms with an adequate height, adapted transport that allows access for the people accompanying them and accessibility aids at the main stops) and the existence of, and respect for, priority seating reserved for the elderly.

Housing. In general, they consider their homes safe, although they say that sometimes they are afraid of being the victims of swindles and/or robberies in their homes (fraudulent electricity or gas meter readings). They report that there is a lack of knowledge of public subsidies for the rehabilitation and adaptation of housing, and highlight the need to improve information on these aspects, together with a simplification of the procedures, since many old homes need to be adapted (installation of a lift, ramps and replacing the bathtub with a shower). They consider it important to draw up a list of elderly persons living alone, in order to provide them with information on social benefits and resources. With regard to day centres for the elderly, they consider that they have adequate conditions, but they do not see them as pleasant places to live in, preferring to stay at home with the aid they might need. In addition, they highlight the shortage of places in public centres (centres that are owned by the State and have public funding) and the excessive price of private centres (centres belong to private individuals that are financed by their own private capital).

Social participation. They underline their participation in cultural, sports, educational and leisure activities in the city. They highly appreciate the existence of activities organized for people their age, with reduced fees and flexible timetables, since most of them have family responsibilities to attend to. They consider it important that the activities be carried out in places in the city centre or in areas served by public transport, in order to make them more easily accessible. They also deem it essential to offer more activities related to sports, cognitive stimulation and nutrition, as a means to help them develop healthy habits. With regard to cultural and leisure activities, they ask for a greater offering of specific activities for their age group, stressing the importance of promoting the use of resources such as theatres and/or cinemas that have suitable facilities.

Civic participation and employment. Elderly persons who participate in volunteer activities are satisfied with their work, although they indicate that this participation is reduced because they are unaware of associations in the city and the volunteer programmers that are carried out. The people who form part of the governing bodies of the associations for the elderly consider that their work is not recognized, which leads to a lower motivation to collaborate in community work. They propose greater coordination with the city institutions that provide services for the elderly.

Communication and information. The elderly value verbal communication as the ideal means of communication (by telephone or information stands) and stress the need to provide information in a timely manner so that it is useful. They drew attention to the reduced usefulness of the press, as it usually reports on activities and events once they have taken place. They show a scarce use of technologies to obtain information, and although they say they have little interest in Information and Communication Technologies (ICT), they call for training in this respect, a large number of elderly persons have to teach themselves how to use them as their use is often needed.

Respect and social inclusion. They state that they do not feel discriminated against because of their age, and that they are treated well by the staff of the Public Administrations and the businesses in the city. They state that their opinions are taken into account in family decisions, and in general feel that taking care of their grandchildren is gratifying, although sometimes this task is a little too much for them and prevents them from being able to spend time on other activities. Likewise, some of them point out that it is difficult to share time and activities with young people, and therefore call for the promotion of intergenerational activities.

Community support and health services. In general, the elderly appreciate the way they are treated by the Municipal Social Services and at the city’s health centres, including both in primary care and hospitals. However, they express displeasure at the excessive delay in scheduling medical appointments and in granting dependency allowances. They underline the importance of establishing a neighborhood psychological advice and guidance service for the elderly, as well as greater visibility and information concerning healthcare resources. They propose that elderly persons living alone or with a certain degree of dependency should have automatic access to tele-help and tele-care services, as they sometimes find it difficult to apply for them. They also consider it necessary to increase home care by offering a comprehensive service that meets all, not just their, medical needs, thereby favoring the possibility of continuing to live in their homes.

### 3.2. Quantitative

As can be seen in Table 2, of the 203 participants in the quantitative study, 55.2% are women and 44.8% are men, and the sample is practically balanced with respect to the two age groups. Most of them (69.5%) live with someone else, the data showing that in most cases they live with their spouse (67.4%) and, to a lesser extent, with their children (19.1%), friends or family caregivers (13.5%). A t-test was used to determine if there were significant differences between the age-friendly city domains means.

Table 3 and Table 4 show the mean score for each of the domains of an age-friendly city and elderly persons’ life satisfaction, taking into account the age cohort variable (Table 3) and whether they live alone or with someone else (Table 4). The data indicate that there are no significant differences in the score obtained in any city domain between the different groups of elderly persons.

Table 5 offers the results regarding the relationship between life satisfaction and the domains of an age-friendly city, taking into account the age cohort variables and whether they live alone or with someone else. It is observed that in the group of 60–74-year-olds, and in the group of those living with someone else, all the domains correlate positively with life satisfaction. In contrast, in the group over 75 years of age, only the domains of outdoor spaces and buildings, community support and health services and transportation show a significant correlation with life satisfaction. Finally, in the group of elderly persons who live alone, this correlation is present in the domains of outdoor spaces and buildings, community support and health services and housing.

The regression analysis (Table 5) indicates that, in all groups, the community support and health services domain is significantly associated with life satisfaction, whereas other domains that are also associated with life satisfaction in a significant way, such as social participation and outdoor spaces and buildings, are only found in the group of elderly persons who live with someone else. 

## 4. Discussion

The results of the study conducted through the focus groups show that, generally speaking, elderly persons feel comfortable in the city of Castelló. The city is considered safe, which encourages them to go out for a walk, and they therefore appreciate the green areas in the city. Public transportation is considered to operate adequately, according to their assessment of the state of the vehicles and the information provided on routes and timetables. In the same way, they consider the conditions of their homes to be adequate and most of them feel integrated, respected and valued in their family environment. They are also satisfied with the care they receive in public centres, especially health centres.

However, some complaints are made about certain aspects and services, and they consider that some characteristics of the city should be improved in order to adapt to the needs of the elderly persons who live there. Some of the aspects they mention that stand out most include the need to improve the information provided about services aimed at favoring the independence and autonomy of the elderly, which make it possible for them to continue to live in their own home for a longer time. Another aspect to be improved is the state of the pavements, to make it easier for people with reduced mobility to move around the city. In this respect, they emphasize the need to take steps to stop terraces or other elements that hinder circulation from occupying the pavement, as well as restricting the use of bicycles on pavements, which causes a certain feeling of insecurity due to the risk of being run over. With respect to social and civic participation, they call for a greater offer of activities specifically targeted at their age group, and with flexible timetables so as to allow them to attend to family obligations.

The quantitative data presented in Table 3 and Table 4 lead us to the conclusion that, regardless of the age group or whether they live with someone else or not, the domain of outdoor spaces and buildings is the best rated, which coincides with the results of other studies [56]. This domain is seen as essential to ensuring mobility and independence among the elderly. 

It is interesting to note that the domain that is rated second best is transportation, which, following the WHO [14], largely determines social participation, an area which has obtained a low score, possibly, as pointed out in the qualitative study, due to the need to offer a wider range of specific activities that are suited to their age and with more flexible timetables.

The area rated the lowest by all the groups of elderly persons is that of civic participation and employment, as in other studies [57]. This is probably due to the lack of information that the elderly claim they receive in this respect. We therefore consider it essential to foster their contribution to the community, since the degree of participation of elderly persons in the social, civic and economic life of the city is closely related to their experience of inclusion [14].

These data, referring to the first aim of our study, enable us to determine that Castelló is considered an age-friendly city, but the average scores obtained also indicate that it is necessary to continue working on the different domains in order to further adapt the city to the needs of the elderly.

This is important because, as the WHO [14] indicates, these eight aspects of city life overlap and interact. Respect and social inclusion are reflected in the accessibility of the buildings and spaces and in the range of opportunities that the city offers to older people for social participation, entertainment or employment. Social participation, in turn, influences social inclusion, as well as access to information. Housing affects the need for community support services, while social, civic and economic participation partly depend on the accessibility and safety of outdoor spaces and public buildings. Transportation and communication and information particularly interact with the other areas: without transportation or adequate means of obtaining information to allow people to meet and connect, other urban facilities and services that could support active ageing are simply inaccessible. There is, therefore, a call for coordinated polity action.

With regard to the assessment of life satisfaction, all groups show a good level of satisfaction. If we take into account the age cohort variable (Table 3), although there are no significant differences, the average is higher in the group of the oldest citizens, possibly, as can be seen from the qualitative analysis, due to the fact that some of those aged between 60 and 74 are overloaded with activities because they are still working, in addition to having the twofold obligation of caring for their grandchildren and, in some instances, their elderly parents. On the other hand, the mean level of life satisfaction of elderly persons who live alone is slightly higher than the mean for those living with someone else (Table 4)—a somewhat striking result that coincides with the results of other studies [58].

With respect to the relationship between life satisfaction and the specific domains of an age-friendly city (Table 5), taking into account the different age cohorts (second aim), the data show clear dissimilarities in the influence of the different settings of the environment on life satisfaction, like other studies that have taken this same variable into account [59,60]. These differences may be due to aspects linked to the theory of selectivity [61], which establishes that the perception of future time (open-limited) influences the establishment of the type of goals that people set themselves. Thus, for the group of younger elderly persons, all areas are relevant to feeling satisfied with life, coinciding with the results obtained by Park & Lee [49]. In the group of the more elderly, however, we observe that the three domains that only significantly relate to life satisfaction are areas that refer more to individual aspects, such as the spaces they use, transportation and the community support and health services available to them.

Yet, if we take into account whether the elderly persons live alone or with someone else (third aim), the results show differences in the domains that are considered important when it comes to feeling satisfied with life. Thus, for the group of elderly persons who live with someone else, all areas are significant, unlike those living alone, who grant greater importance to more individual aspects such as outdoor spaces, community support and health services and housing.

We therefore found that outdoor spaces and buildings and community support and health services are the domains that are significantly related to life satisfaction for all the groups of elderly persons.

The data from the regression study (Table 5) indicate that the elderly in the different groups are more satisfied with life if they have adequate community support and health services, which leads us to establish this domain as a possible predictor of our elderly citizens’ life satisfaction. This is in line with the WHO [14], which qualifies this area as vital for maintaining health and independence in the community and indicates that elderly persons, regardless of the context and the experiences they have had, express a clear desire for basic health services and income. Likewise, they also consider that the cost of healthcare is very high and express a common desire for care at an affordable cost.

In the regression analysis of the group that live with someone else, unlike the rest of the groups, the variables social participation and outdoor spaces and buildings also appear as predictors of life satisfaction, possibly due to the fact that living with someone else makes you more likely to go out for a walk or sign up for activities, i.e., carry out more physical activity, than those who live alone [62]. Consequently, it would be important to establish specific actions to increase the social participation and activity of elderly persons who live alone.

Taking into account these results, as well as those obtained in similar studies consulted [49,56,57,58,59,60], and after reviewing various strategy plans [63,64] based on international gerontological strategies and plans, some recommendations for developing policies that foster age-friendly environments, would be: to facilitate the involvement of older people in city decision-making; promote the empowerment of older people through the promotion of autonomy; to disseminate a realistic picture of ageing people to all generations and to combat discrimination; ensuring the security and social inclusion of older people; encourage a positive transition from working life to retirement; value the transfer of care and support provided by older people; preparing homes and environments that can be enjoyed throughout life, promoting permanence in their surroundings; promote healthy ageing; incorporate lifelong learning into society; promote volunteering and social participation; and foster intergenerational solidarity.

## 5. Conclusions

The overall ageing of the urban population demands that our cities implement approaches that are better adapted to the elderly [42]. Age-friendly cities are fundamental to achieving places where elderly persons are actively involved, valued and have the support of infrastructure and services that are effectively adapted to their needs [65]. A safe, adequate and barrier-free living environment can help them adapt to age-related changes and positively influence their well-being [66], otherwise they may experience maladjustment and their subjective well-being could be impaired [23].

In this sense, the gerontologists who have tried to determine to what extent the eight domains proposed by WHO are adequate have confirmed the consistency of the domains with empirical research on the environmental and social factors associated with well-being in older age [15,20], with some slight divergences. The area of respect and social inclusion has been the subject of criticism [20], who regard it as a basic value of the initiative rather than as a separate domain. Instead, they propose that variables of economic inequality and social disorder that lead to social exclusion be part of a new domain of social environment. Overall, the Vancouver Protocol does not give adequate coverage to social networks and social support and protection from harm (physical safety and crime protection). Harm protection is featured in items within the WHO domains of outdoor spaces and buildings, transportation and housing, however, projects such as those developed in Toronto or Montreal in 2013 have identified this as a distinct domain. One aspect that has been criticized is that the domain of social participation did not specifically include unstructured contact with family, friends and neighbors, which is considered as an age-friendly community feature. In addition, some initiatives have shown that some domains are less relevant in less developed countries, such as the availability of different housing options or different health services. Therefore, the domains established by WHO are supported by gerontological research, but with some additions and refinements in certain places.

Due to this, it is important that the studies conducted on contextual factors and their effects on elderly persons’ well-being are approached from a life cycle perspective, which proposes that ageing processes and experiences may be different depending on the place and time in history in which they are living. Age-friendly environments may, therefore, be universally beneficial, but their specific effects may vary from one culture or society to another [60,67,68]. Similarly, among elderly persons living in the same city or community, it is essential to take into account their sociodemographic differences (such as the age cohort, or whether they live alone or with someone else). It is also necessary to accept that not all elderly persons have the same characteristics, to establish specific measures to meet the needs of different groups.

Therefore, we stress the importance of conducting studies with a mixed methodology in the future, since qualitative data help to understand and interpret quantitative data, and can provide us with a better understanding of the phenomenon under study [69]. That is, policies that take into account the longevity of society, to encourage the autonomy and independence of older people by promoting permanence in their environments, and to guarantee their participation and social collaboration as an important collective in the construction of a welfare society.

## 6. Limitations

Our study has a number of limitations. Although the AFCQ has adequate reliability, it needs to be improved. A longitudinal study should be conducted to show, e.g., through sequential comparison between nested models, that the scale is invariable over time. Moreover, this would provide further confirmation of the relationship between life satisfaction and predictor variables. Another shortcoming is the measurement of life satisfaction with a single item rather than a scale, as the assessment of life satisfaction could be influenced by the state of mind of the elderly during the survey; the use of a scale could, therefore, affect the results of the study.

With this study, we intend to contribute to the evaluation and identification of those contextual factors that influence elderly persons’ life satisfaction, so that it may serve as an aid to establish policies that strive to build and improve age-friendly environments.

## Figures and Tables

**Table 1:** Qualitative study participants: groups of elderly persons.

Individual Characteristics	*n* = 66	%
Sex		
Female	35	53.1
Male	31	46.9
Age		
60–74	39	59
≥75	27	41
Socio-economic status		
Low	48	72.7
Middle	18	23.7
Educational level		
Primary school	30	45.5
Secondary school	14	21.2
University studies	32	33.3
Lives with someone		
No	12	18.2
Yes	54	81.8
Who do you live with?		
Husband or wife	39	72.2
Son or daughter	13	24
Other (parents, friends, caregivers, etc.)	2	3.8

**Table 2:** Quantitative study participants.

Individual Characteristics	*n* = 203	%
Sex		
Male	91	44.8
Female	112	55.2
Age-group years		
60–74	109	53.7
≥75	94	46.3
Lives with someone		
No	62	30.5
Yes	141	69.5
Who do you live with?		
Husband or wife	95	67.4
Son or daughter	27	19.1
Others (parents, friends, caregivers, etc.)	19	13.5

**Table 3:** Mean scores of age-friendly city domains and life satisfaction of participants based on age.

Domain	60–74 (*n* = 109)		≥75 (*n* = 94)		*p* Value
	Mean	SD	Mean	SD	
Outdoor spaces and buildings	3.66	0.79	3.73	0.84	0.59
Transportation	3.36	0.59	3.28	0.62	0.38
Housing	3.04	0.61	3.10	0.72	0.49
Respect and social inclusion	3.09	0.69	3.05	0.66	0.70
Social participation	2.92	0.87	3.07	0.73	0.19
Communication and information	3.06	0.71	3.11	0.70	0.57
Civic participation and employment	2.71	0.57	2.67	0.58	0.64
Community support and health services	2.95	0.80	3.02	0.82	0.57
Life satisfaction	6.10	1.37	6.45	1.68	0.10

**Table 4:** Mean scores of age-friendly city domains and life satisfaction of participants based on whom they live with.

Domain	Living Alone (*n* = 62)		Living with Others (*n* = 141)		*p* Value
	Mean	SD	Mean	SD	
Outdoor spaces and buildings	3.62	0.85	3.73	0.80	0.40
Transportation	3.24	0.55	3.36	0.62	0.17
Housing	3.01	0.76	3.09	0.61	0.45
Respect and social inclusion	3.01	0.71	3.10	0.66	0.42
Social participation	2.96	0.87	3.00	0.79	0.79
Communication and information	3.12	0.72	3.07	0.70	0.59
Civic participation and employment	2.62	0.58	2.72	0.57	0.25
Community support and health services	2.99	0.79	3.72	0.81	0.97
Life satisfaction	6.40	1.60	6.20	1.49	0.38

**Table 5:** Correlations and results from the regression analyses of satisfaction and age-friendly city domains according to age and whether they live alone or not.

Domain	60–74 Years *n* = 109		≥75 Years *n* = 94		Living Alone *n* = 62		Living with Others *n* = 141	
		β ^a^		β ^a^		β ^a^		β ^a^
Outdoor spaces and buildings	0.31 **	0.184	0.31 **	0.238	0.32 *	0.245	0.30 **	0.188 *
Transportation	0.25 **	0.084	0.23 *	0.191	0.18	0.049	0.26 **	0.177
Housing	0.25 **	0.001	0.12	−0.146	0.22 *	0.075	0.16 *	−0.162
Respect and social inclusion	0.31 **	0.140	0.05	−0.116	0.11	−0.130	0.21 **	0.041
Social participation	0.29 **	0.044	0.20	0.189	0.14	0.147	0.30 **	0.214 *
Communication and information	0.32 **	−0.001	0.14	−0.057	0.10	−0.177	0.28 **	0.038
Civic participation and employment	0.26 **	−0.070	0.02	−0.183	0.09	−0.075	0.15 *	−0.158
Community support and health services	0.39 **	0.260 *	0.26 *	0.298 **	0.30 **	0.338 *	0.33 **	0.228 *

* p < 0.05; ** p < 0.01. ^a^ Standardised beta coefficients of predictors of life satisfaction.
